# The Barriers and Facilitators of Different Stakeholders When Deprescribing Benzodiazepine Receptor Agonists in Older Patients—A Systematic Review

**DOI:** 10.3390/metabo11040254

**Published:** 2021-04-20

**Authors:** Anja Fog Rasmussen, Sarah Sonne Poulsen, Lykke Ida Kaas Oldenburg, Charlotte Vermehren

**Affiliations:** 1 Department of Pharmacy, Faculty of Health and Medical Sciences, University of Copenhagen, Universitetsparken 2, 2100 Copenhagen, Denmark; wqv740@alumni.ku.dk (A.F.R.); nwh364@alumni.ku.dk (S.S.P.); lykke.ida.kaas.oldenburg@regionh.dk (L.I.K.O.); 2 Department of Clinical Pharmacology, University Hospital Copenhagen, Bispebjerg Bakke 23, 2400 Copenhagen, Denmark

**Keywords:** deprescribing, barriers, facilitators, older patients, benzodiazepines, Z-drugs, BZRA

## Abstract

Treatment of older patients with benzodiazepines and Z-drugs (BZRA) is associated with an increased risk of side effects. However, this treatment is still used among these patients. Deprescribing can be a tool to reduce inappropriate medication. This review aims to identify and compare barriers and facilitators of stakeholders involved in BZRA deprescribing in older patients and uncover potential gaps in the research field. The search was conducted in PubMed, EMBASE, PsycINFO, and Cochrane Library. Ten articles based on qualitative data on BZRA deprescribing in older patients (≥65 years) published between 2005–2020 were included. Six articles referred to patients as stakeholders, two referred to physicians, and one to nurses and caregivers, respectively, indicating a need for more studies in the field. More barriers than facilitators were identified. Important findings were the patient willingness to deprescribe BZRA compared to physicians, who did not mention deprescribing to patients due to barriers such as expected patient resistance. Nurses mentioned barriers like lack of knowledge and the feeling that their options were not valued by physicians; education was found to be a shared deprescribing facilitator among the stakeholders. Being aware of deprescribing barriers and facilitators can be helpful in future successful deprescribing interventions.

## 1. Introduction

Benzodiazepines are used in the treatment of a variety of conditions, including insomnia and anxiety. Closely related so-called Z-drugs (zopiclone, eszopiclone, zolpidem, and zaleplon) are also used in the treatment of insomnia. For the purpose of this review, the term Benzodiazepine Receptor Agonist (BZRA) is used as a combined term for benzodiazepines and Z-drugs. The sedative effect of BZRA decreases within days to weeks [1,2,3]. Pharmacological treatment with BZRA in older patients is associated with an increased risk of falls, balance issues, drowsiness, cognitive impairment, memory disorders, functional impairment, and physical dependence [2,4]. In this review, older patients are defined as patients at the age of 65 years or older. Age-related changes in pharmacokinetics and pharmacodynamics might be some of the causes of the increased risk of harm in older patients [4].

Despite awareness of the increased risk of harm, prescription of benzodiazepines to older patients still takes place [5,6]. Benzodiazepine users tend to be older patients and in general benzodiazepine consumption increase with increasing age [5,6]. The use of BZRA may be indicated in certain circumstances [7]. However, the majority of BZRA prescriptions for the older people are considered inappropriate [7]. Deprescribing has been suggested as a tool to reduce or eliminate inappropriate medications. Deprescribing is defined as the “planned and supervised process of dose reduction or stopping of medication that might be causing harm or no longer providing benefit” [2]. Numerous recommendations for deprescribing exist for inappropriate use of drugs, e.g., a Canadian Evidence-based Clinical Practice Guideline recommends deprescribing of BZRA for insomnia among people aged 65 years or older, regardless of the duration of treatment [2]. Beers Criteria recommend that the use of BZRA in older patients should be avoided, with some exceptions for benzodiazepines such as treatment for seizure disorders [7].

Several stakeholders can be involved in deprescribing BZRA in older patients such as healthcare personnel and patients [4,8]. To our knowledge, no systematic review exists with the main focus of exploring and comparing the barriers and facilitators of stakeholders towards deprescribing BZRA in older patients.

### Objective

This review aims to identify and compare the barriers and facilitators towards deprescribing held by potential stakeholders involved in the decision making of BZRA deprescribing in older patients. In addition, this review aims to uncover potential gaps in the research field, such as a lack of research or poor-quality research on individual stakeholders involved in this decision making.

## 2. Methods

The protocol of the review was registered at the PROSPERO international database of prospectively registered systematic reviews (CRD42020200516). The eligibility criteria were attitudes including barriers and facilitators towards deprescribing BZRA. Qualitative studies were sought to provide insight into the attitudes of relevant stakeholders by capturing life experiences and exploring attitudes. Mixed method studies were also included if the qualitative data were distinguishable from the quantitative data. Only published and English written articles were included. To capture attitudes of different stakeholders towards deprescribing of BZRA in older patients, participants sought to be included were either older patients in treatment with BZRA and/or stakeholders involved in decision making on BZRA deprescribing in older patients. Stakeholders are defined in this review as persons who can influence the decision making towards deprescribing for older patients.

The search strategy was developed by the two authors (A.F.R. and S.S.P.) and was debated with a Subject Specialist at Copenhagen University Library. The elements of Population, Phenomenon of interest, and Context (PICo) [9] were used to identify search terms in accordance with the objective. The population used for our review was older patients. The phenomenon of interest was deprescribing and the context was BZRA.

MEDLINE (via PubMed interface, 1946 onwards), EMBASE (via OVID interface, 1974 onwards), PsycINFO (via EBSCO interface, 1887 onwards), and Cochrane Library (via Wiley interface, 1992 onwards) [10] were searched during 15 May 2020 by the two authors. The time limitation was from 1 January 2005 to 15 May 2020.

Results from the literature search were uploaded to the reference manager (EndNote) [11] where duplicate citations were excluded. The two authors independently screened titles, abstracts, and full-text articles of the search results based on the eligibility criteria. When there was any uncertainty while screening titles, the articles were included for abstract screening. When there was any uncertainty while screening abstracts, the articles were included for full-text reading. Reasons for exclusions were discussed between the two authors and for the full-text screening, reasons for exclusions were recorded. Any disagreements in the study selection after the full-text screening were first discussed between the two authors, and then discussed with the co-authors.

Two authors (A.F.R. and S.S.P.) conducted data extraction independently. The data extraction was pilot tested on three of the included articles. Disagreements were first discussed between the two authors, and then they consulted the co-authors. Predetermined data outcomes of the review were barriers and facilitators. Barriers were defined as views of the stakeholders which can create a challenge for deprescribing of BZRA in older patients. Facilitators were defined as views of the stakeholders which will facilitate deprescribing of BZRA in older patients. Initially a third data outcome was included, which was attitudes. During data analysis, attitudes was eliminated as a data outcome. Data outcomes in each article were extracted using content analysis to quantify the amount of content into themes combined with a more qualitative thematic approach [12]. The data outcomes were extracted from the result section of the articles using the NVivo version 12 software program for qualitative research [13]. The extractions for each outcome were divided into groups of stakeholders. Two authors (A.F.R. and S.S.P.) synthesized themes and subthemes independently. The themes of each outcome were divided into (1) Individual and (2) Shared. The individual themes were solely present in one group of stakeholders. The shared themes were present for two or more groups of stakeholders.

Consolidated Criteria for Reporting Qualitative Research (COREQ) was used in the quality assessment of the included articles [14]. Two authors (A.F.R. and S.S.P.) conducted the quality assessment independently and discussed disagreements.

## 3. Results

### 3.1. Literature Search

**Figure 1:**
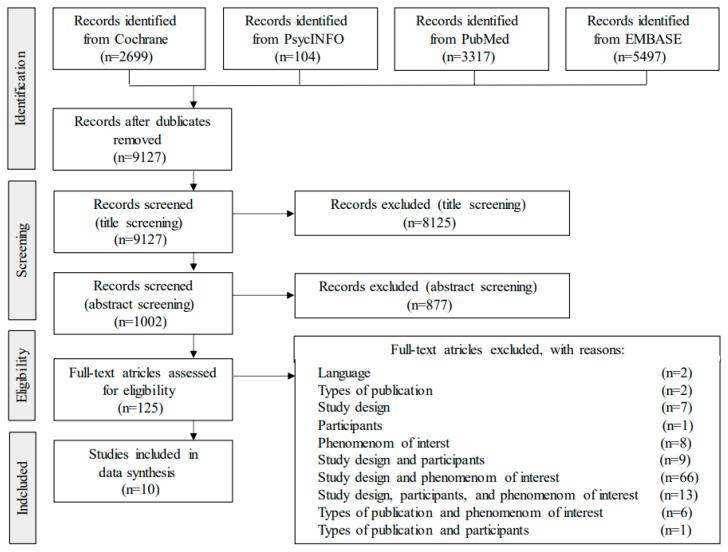
PRISMA flow. The search strategy and selection process for included articles for the review.

The literature search resulted in a total of 9127 records after removing duplicates. 8125 records were excluded from screening of titles. Additionally, 877 records were excluded from screening of abstracts. 125 full-text articles were assessed for eligibility and 10 articles were eligible for the purpose of the review (Figure 1).

### 3.2. Description of the Articles

The qualitative part of the studies yielded barriers and facilitators towards deprescribing of BZRA in older patients from four different stakeholders. The largest quantity of the articles examined barriers and facilitators of patients (Table 1). The remaining articles examined physicians, nurses, and caregivers. The total number of stakeholders for all included articles was 230 patients, 43 physicians, 33 nurses, and 17 caregivers.

### 3.3. Quality Assessment

Reporting varied with articles reporting an average of 17 (range 15–20) of the 32 items in the COREQ checklist. The first domain has the lowest rate of reporting, where no article reported the gender of the researcher or the three items regarding the relationship with participants. The second domain regarding study design has a higher rate of reporting, where the two items, methodological orientation and sample size, were reported in all included articles. The two items: The presence of non-participants and Transcripts returned to the participants for comments, were not reported in any of the articles and only one article reported the item Interview guide pilot tested. The highest rate of reporting was found in domain three, where the items Derivation of themes, Data and consistent findings, Clarification of major themes, and Minor themes was reported in all the articles. Only one article reported the items Description of the coding tree and Patients providing feedback on findings.

### 3.4. Barriers and Facilitators Are Shared between the Stakeholders

Some of the identified barriers and facilitators were presented across the different groups of stakeholders. The themes derived from the content analysis are presented in Table 2 and Table 3. These barriers and facilitators will be referred to as shared (Table 2). Caregivers were not represented as a separate group of stakeholders in the tables (Table 2 and Table 3) due to limited data. Only one facilitator was identified for the caregivers, i.e., Perception of benzodiazepines being of low value due to side effects (Table 2). In the table, the number of times the theme is mentioned in the articles is presented in cursive after the theme.

### 3.5. Shared Barriers towards Deprescribing BZRA

The shared barriers were categorized into the following themes: (1) Effectiveness of treatment, (2) BZRA provide comfort, (3) BZRA do no harm, (4) Concern about withdrawal symptoms, (5) Ageism, (6) Lack of knowledge, and (7) Working environment and procedures (Table 2).

Finding the BZRA treatment effective was a barrier shared between patients, physicians, and nurses [16,17,19,20,22]. Likewise, some patients and nurses believe that the BZRA treatment provides the patient with a feeling of comfort, creating a view of continued use as being necessary [8,15,16,22].

Some patients and physicians believe that continued treatment with BZRA causes no harm to the patient [17,18,19,20]. This was mentioned by some of the physicians, while others believed the advantages outweighed the problems [20]. Some physicians felt that deprescribing lead to unnecessary suffering for the patients [20], which was shared with the patients who expressed concern about withdrawal symptoms [8,17,18,20]. Physicians and patients also agreed upon ageism as a barrier [8,16,17,20]. One out of thirty three physicians found no need to bother with deprescribing while another was more lenient in continuing treatment due to the patients´ old age [20]. Some patients found that their physician encourages them not to worry about continuing treatment due to their old age [17]. However, some of the patients did not worry about continuing treatment due to their age [8,16,17].

Generally, a lack of knowledge or incorrect knowledge was identified for both patients and nurses [8,16,19,22]. Both patients and nurses lack knowledge of the side effects of BZRA treatment, which created a barrier when a side effect was not linked to the treatment [8,19,22]. Additionally, patients had no or little knowledge of alternative treatments and a lack of insight about the dependence related to BZRA use [16,19]. Nurses reported difficulty in differentiation of generic products, and they were concerned that it could result in the patients getting their medication twice.

The working environment and procedures were mentioned by both physicians and nurses [20,21,22]. Time was mentioned by the nurses referring to a shortage of staff and overwhelming work pressure [22]. Some of the physicians complained that a lack of time in consultation and high workloads created a barrier [20,21]. Virtually none of the 33 physicians in the study by Cook et al. [20] considered deprescribing of BZRA to be a priority and found other problems to be more important to address in the short consultation time [20]. Physicians stated that they would not risk losing patients due to BZRA deprescribing, as they believed that the patient would find another physician to prescribe BZRA [20]. In contrast, one physician quotation from the study by Subelj et al. [21] reported that the physician would not prescribe unnecessary medication in fear of losing patients. In the study by Cook et al. some physicians reported a lack of guidelines on tapering BZRA, alternative treatments, and addressing patients’ concerns [20]. No assessment of patients’ need for continued BZRA treatment nor evaluation of the treatment was performed according to the nurses [22]. A nurse mentioned that observations on patients’ need for continued treatment were not recorded or documented [22]. Some nurses mentioned that their work was more task-oriented compared to patient-oriented work. The nurses needed to have all residents in bed and completed medication rounds before the night shift started, which enhances BZRA use according to the staff. Another barrier was discouragement and resistance from colleagues [22].

### 3.6. Shared Facilitators towards Deprescribing BZRA

The shared facilitators were categorized into the following themes: (1) Education, (2) Improving cooperation, (3) Patient-motivation from healthcare personnel, and (4) Cessation of side effects.

A facilitator shared between patients, physicians, and nurses was education [17,21,22]. Some nurses mentioned education in sleep hygiene as being important [22]. Some patients found the education tool (a deprescribing brochure on benzodiazepines) to be useful [17]. Physicians expressed a need for education from psychiatrists [21]. Improving cooperation was a facilitator shared between physicians and nurses [21,22]. In addition, physicians mentioned a better cooperation with psychiatrists, e.g., direct communication with psychiatrists and access to psychiatrists [21]. Some nurses mentioned improving cooperation and communication between day and night shifts, as night shift staff observe patients’ sleep and can contribute to the discussion of patients’ need for BZRA [22].

A facilitator shared between patients and physicians was motivation [8,17,21]. Motivation of patients by physicians was mentioned in the context of options for alternative treatments [21]. In the context of motivation from other stakeholders, the study by Chen et al. [8] presented one patient quotation explaining that motivation of a pharmacist could facilitate deprescribing. Caregivers and patients mentioned cessation of side effects [17,23]. The caregivers felt that treatment with BZRA was undervalued, as patients were prevented from carrying out daily activities due to side effects [23]. In the study by Martin et al. [17], all patients were more willing to try deprescribing, once a correlation between their complaints and side effects of their BZRA treatment was explained.

### 3.7. Individual Barriers towards Deprescribing BZRA

The patient barrier towards deprescribing BZRA mentioned most times was dependence [15,16,19]. Patients relied on BZRA for comfort [8,15,17,19]. Likewise, patients felt they were unable to sleep without BZRA [15,17,19]. Other barriers reported by the patients was experiences of discouragement from their physician [17]. Patients expressed that a barrier was previous reassurance from physicians of the safety or the necessity of the treatment [17]. Patients believed that their physician approved the treatment because the physicians did not mention deprescribing or potential harm in continuing treatment [17,19]. A minor theme presented in the study by Heser et al. [16] was that lack of support from relatives influenced the patient.

The physicians expected that the patients would be resistant [20]. In the context of this barrier, the physician most often mentioned that patient resistance was time-consuming, which created another barrier due to limited consultation time. Other contexts of patient resistance were that physicians mentioned that dealing with patient resistance was not worth the fight and could cause the physician to lose patients [20]. Physicians mentioned a reluctance to deprescribe based on what they believed was stable or functioning patients [20,21].

Some nurses felt an unequal balance of power between themselves and the physicians [22]. The nurses felt their opinion was not considered or valued by the physician. Some nurses felt powerless to challenge the prescription or hesitant to contradict the physician. A barrier that was shared among the 33 included nurses was that they felt BZRA use should not be avoided. Most nurses did not have a critical view of BZRA use [22].

### 3.8. Individual Facilitators towards Deprescribing BZRA

Most of the five patients in the study by Chen et al. [8] wanted to decrease their use of BZRA. This was supported in the study by William et al. [19], which reported that many of the 17 participating patients were willing to deprescribe the BZRA treatment. Patients once they had time to consider the suggestion of deprescribing were willing to try this option [8,17].

The physicians in the study by Cook et al. [20] in general agreed with guidelines for only prescribing BZRA on a short-term basis. The physicians in the study by Subelj et al. [21] agreed that BZRA should be used on a short-term basis.

Several nurses expressed a willingness to become more involved in the use and deprescribing of BZRA [22]. One nurse expressed that there was more involvement in BZRA use among nursing home residents. Systematic work procedures to address the problem of continued BZRA treatment were presented as a possible facilitator. One nurse believed that the nurses could play a key role in the use of BZRA [22].

## 4. Discussion

### 4.1. Stakeholders Involved in BZRA Deprescribing: Individual and Shared Barriers and Facilitators

This review identified and compared barriers and facilitators of four groups of stakeholders: patients, physicians, nurses, and caregivers. In the review by Ng et al. [4] physicians and patients were most often targeted in interventions towards deprescribing BZRA in older patients. Additionally, relatives, pharmacists, and professional caregivers were often not targeted in the interventions [4]. The same applies in our review, as data primarily explored patients and secondly physicians. No articles exploring pharmacists´ attitudes were identified, which shows gaps in the research. Also, the caregivers included both relatives of the patients and professional caregivers, therefore, the review could not separate the two.

Individual barriers and facilitators of the stakeholder groups were identified. Some barriers and facilitators were shared and therefore were possible to address in general in the context of an intervention study. Meanwhile, other barriers and facilitators were solely necessary to address in the given group of stakeholders in the context of an intervention. In this review, more barriers than facilitators were identified. Barriers and facilitators identified in this review are illustrated in Table 2 and Table 3. In the following, the main findings will be discussed.

### 4.2. Stakeholder Attitudes Influence the Willingness of Patients to Deprescribe BZRA

An important identified facilitator was patients’ willingness to deprescribe BZRA treatment [8,19], which is in contrast to the physician’s perception of patient resistance to deprescribing [19]. As far as we know, this has not been reported by others. Though most patients were willing to attempt deprescribing, several barriers were identified (Table 2 and Table 3). In agreement, a review by Ng et al. [4] exploring deprescribing interventions of BZRA in older patients also discovered barriers such as lack of knowledge, dependence, and patients’ confidence in their physician´s approval to continue the treatment. The review reported that interventions targeted at patients were influenced by the perceptions and beliefs of the physicians, and other healthcare providers [4]. Our data show that physicians influenced the patients’ attitudes by discouraging deprescribing, lack of support, or reassurance of the safety or the necessity of the treatment. Also, if the physicians did not mention deprescribing of BZRA in consultations with the patients, some patients interpreted this as physicians’ acceptance towards continuing the treatment [17,19]. Our data on physicians’ barriers suggested that in some cases, this interpretation might be correct. Some physicians directly or indirectly expressed that they were more lenient with their prescribing of BZRA to older patients and, therefore, might not mention deprescribing in consultations. Physicians mentioned reluctance to deprescribe due to what they believed was stable or functioning patients [20,21]. Yet, in this study, barriers to contradict the patients’ interpretation of physicians’ attitudes to deprescribing were identified. Physicians mentioned that deprescribing was not suggested to patients due to a lack of time, high workload, and prioritizing [20,21]. In addition to this, our data suggest that pharmacists and patients’ relatives influence the attitudes of the patients as well and thereby affect the process of deprescribing [8,16]. In conclusion, these data show a need for increased communication and shared decision-making between physicians and patients in order to optimize medication according to patients’ preferences. Recent reviews by Reeve et al. [24] and Scott et al. [25] on deprescribing stressed the importance of patient-involvement and shared decision-making. The creation of interventions with a focus on patient involvement and shared decision-making can be difficult when evidence on the barriers and facilitators of stakeholders involved are lacking or non-existing.

### 4.3. Nurses Call for Education and Support to Facilitate BZRA Deprescribing

In a study by Kua et al. [26] involving 17 participants (4 physicians, 4 pharmacists, and 9 nurses), it was shown that nurses were able to contribute with information about side effects [26]. In the study, the importance of team communication when deprescribing was pointed out. In contrast, our data showed that nurses did not have a critical view on the use of BZRA and expressed the use as necessary [22] (Table 3), suggesting that a change in attitude is necessary to achieve successful deprescribing interventions. Anthierens et al. [22] showed that the nurses were willing to get more involved in the appropriate use of BZRA, e.g., through education and the use of an interdisciplinary team, respectively (Table 3). However, their work environment prevented a focus on evaluation and reflection on the patients’ BZRA use. Also, a lack of influence on the physicians’ decisions due to the unequal balance of power created a barrier preventing the nurses from involving themselves in deprescribing of BZRA [22]. Hence, our data suggested that nurses are an unused source of valuable information in the deprescribing of BZRA in older patients.

### 4.4. Caregivers Facilitate BZRA Deprescribing Due to Observed Side Effects among Patients

Data collected on the caregivers were limited to one article identifying one facilitator to deprescribing BZRA in older patients [23]. The study aimed to identify the most significant factors that impact the perceived value of medication from the perspective of patients and caregivers [23]. Here, caregivers expressed that patients were prevented from carrying out daily activities due to side effects, which resulted in a low value for BZRA use [23]. Chen et al. [8] reported that when the connections between complaints and side effects of the BZRA treatment were explained, the patients were willing to deprescribe. Therefore, our data suggest that caregivers, could be a valuable, yet, unused or unexplored facilitator of deprescribing.

### 4.5. Future Directions for Research

To promote successful interventions more research on the barriers and facilitators towards deprescribing BZRA in older patients is needed. As the evidence on pharmacists, relatives, caregivers, and nurses is non-existing or limited, research on these stakeholders could be favorable to target in future research on the topic. Additionally, further research to investigate the barriers and facilitators of Z-drugs could be beneficial, as only one article included Z-drugs. The reason for the lack of studies on deprescribing of Z-drugs may be due to physicians perceiving Z-drugs as more effective and safer compared to benzodiazepines [27,28]. Generally, the content of the included articles focused more on barriers than on facilitators. The data indicate that future research could benefit from focusing more on facilitators to deprescribing BZRA in older patients.

## 5. Strengths and Limitations

The protocol was specified in advance and registered in an international prospective register of the systematic review. The study design allows for exploring a wide range of possible barriers and facilitators towards deprescribing in older patients and allowing inclusion outcomes that were not expected by the researchers [12]. Limitations include a relatively small number of articles addressing the objective and the unequally in data represented for the stakeholders, which limits the generalizability [12] as more articles are needed for nurses, caregivers, and physicians to make more valid conclusions. In addition, conducting a meta-synthesis on the identified qualitative studies has not been performed, which is a further limitation to the study. Hence, conducting the same study through the application of meta synthesis method to data should be considered.

## 6. Conclusions

This review indicates that the deprescribing process is influenced by the attitudes of different stakeholders i.e., patients, physicians, nurses, and caregivers. Several deprescribing barriers and facilitators were identified. It was found that patients are willing to deprescribe their BZRA treatment, while doctors consider that patients will resist this. In addition, it was shown that nurses and caregivers are an unused source of support in a deprescribing process. However, they demand education regarding the effect and side effects of BZRA. Finally, the results showed a need for shared decision-making between physicians and patients in terms of deciding on BZRA deprescribing. Knowing and being aware of the individual as well as shared barriers and facilitators between the stakeholders can be helpful in conducting future successful deprescribing interventions to ensure appropriate medication of older patients.

## Figures and Tables

**Table 1:** Characteristics of the qualitative studies exploring the barriers and facilitators of different stakeholders towards deprescribing BZRA in older patients.

Stakeholder	No. of Participants	Setting (Country)	Gender %F	Age	BZRA Consumption and Prescribing	Data Collection Method (Analytical Method)	First Author (Year) [Ref. No.]
Patients	12	Other (USA)	100%	65–89	Near daily or daily BZRA use ≥3 months	Semi structured interview (Thematic analysis)	Canham (2014) [15]
Patients	5	Hospital (Canada)	-	79–91	Daily BZRA use ranging from recent initiation to 30 years	Semi structured interview (Constant comparative analysis)	Chen (2010) [8]
Patients	10 *	GP (Germany)	80%	86–96	Nine participants had a chronic BZRA use. One participant non-chronic BZRA use.	Semi structured interview (Content analysis)	Heser (2018) [16]
Patients	21 **	Pharmacy (Canada)	72%	74.6 ± 6.3	Chronic users of BZRA	Semi structured interview (Thematic content analysis)	Martin (2017) [17]
Patients	123 ***	Pharmacy (Canada)	69%	74 ± 6.3	The mean duration of BZRA use was 10 years	Semi structured interview (Content analysis)	Tannenbaum (2014) [18]
Patients	17	GP (Australia)	76%	77 (F), 73 (M) (mean)	Nocturnal BZRA use ranging from 1 year to more than 20 years	Semi structured interview (Constant comparative analysis)	Williams (2016) [19]
Physicians	33	Other (USA)	33%	47 (mean)	Practice characteristics include family medicine, geriatrics, and general internists	Semi structured interview (Narrative analysis)	Cook (2007) [20]
Physicians	10	GP (Slovenia)	20%	****	Five low- and high-prescribing family physicians, respectively. The high prescribers had practiced 18 years on average, which is six years more than of the low prescribers	Semi structured interview (Thematic analysis)	Subelj (2010) [21]
Nurses	33	Nursing home (Belgium)	76%	37 (mean)	All nurses had a bachelor’s degree Mean years of experience was 14	Focus group and semi-structured interview (Thematic analysis)	Anthierens (2009) [22]
Caregivers	17	Other (USA)	82%	22–69	10 caregivers cared for family members, 7 were employed via home care agencies, nursing homes, group homes *****	Focus group (Thematic analysis)	Pickering (2020) [23]

F = female, M = male, GP = General Practice. * The total no. and age of participants in the studies were 52, of which 10 used BZRA. Only qualitative data on the 10 BZRA users are included in the content analysis. ** Mean age and gender is for the 261 total participants, of which 21 participants took part in the qualitative part of the study. *** The total no. of participants was 261 of which qualitative data is presented for 123 participants. **** Mean age of the high-prescribers was almost 10 years higher than that of the low-prescribers. All high prescribers were male. ***** Their tasks including filling pillboxes, administering medications, performing or coordinating associated testing, or communicating with providers. Articles were published between 2007 and 2020. Settings included hospitals, general practices, pharmacies, nursing homes, and others. Some studies recruited participants from postal mailing, word-of-mouth, phone solicitation, advertisements, or research registries. Eight articles were based on studies with a sole focus on benzodiazepines [8,15,17,18,19,20,21,22]. Two articles had a general focus on Potential Inappropriate Medication (PIM) [16,23] but only data regarding benzodiazepines and/or Z-drugs was extracted for the content analysis. Z-drug was included in one of the articles [16]. Patient diagnosis or indication, i.e., insomnia, for the BZRA treatment was specified in one of the articles [19].

**Table 2:** Key themes of shared barriers and facilitators for deprescribing BZRA and frequency of themes for each individual stakeholder.

Shared Barriers			
	Patients	Physicians	Nurses
Themes	Frequency [Ref. No.]	Frequency [Ref. No.]	Frequency [Ref. No.]
Finding BZRA to be an effective treatment	4 [16,17,19]	2 [20]	2 [22]
Finding BZRA provide comfort for the patient	5 [8,15,16]	No data	1 [22]
Conceive that BZRA does not harm the patient	3 [17,18,19]	2 [20]	No data
Concern about withdrawal symptoms	4 [8,17,19]	2 [20]	No data
Ageism: Finding deprescribing BZRA as unnecessary due the age of the patient	3 * [8,16,17]	2 [20]	No data
Lack of knowledge	6 [8,16,19] Including side effects, alternative treatments, dependence	No data	9 [22] Including side effects, effects, sleep in general
Working environment and procedures	No data	10 [20,21] Incl. lack of time, deprescribing not being prioritized, concern about losing patients, lacking strategies to taper, and cooperation with specialists/psychiatrists	14 [22] Incl. lack of time, lacking assessment and observations of the need for continued BZRA treatment, staff shortage, high work pressure, task-oriented work, lack of involvement in medicine
**Shared Facilitators**			
	**Patients**	**Physicians**	**Nurses and Caregivers**
**Themes**	**Frequency [Ref. No.]**	**Frequency [Ref. No.]**	**Frequency [Ref. No.]**
Education	1 [17] Education tool: deprescribing brochure on benzodiazepines	1 [21] Education about psychiatric disorder from psychiatrists	1 [22] Nurses Education about sleep hygiene
Improving cooperation between healthcare personnel	No data	2 [21] Cooperation and clear instructions from psychiatrists	2 [22] Nurses Address sleep problems by interdisciplinary team
Patient-motivation	3 [8,17] Motivation from the physician/pharmacist	1 [21] Physicians providing motivation to the patients	No data
Awareness of side effects	1 [8] Describe BZRA side effects relevant to the patient’ complaints	No data	1 [23] Caregivers Benzodiazepines are of low value due to of their side effects

* Mentioned a 4th time in Martin et al. [17], the patients mention that the physician will not deprescribe due to the patients’ old age.

**Table 3:** Key themes of individual barriers and facilitators for deprescribing BZRA and frequency of theme for each individual stakeholder.

Individual Barriers			
	Patients	Physicians	Nurses
**Theme** **Frequency [ref.no.]**	**Dependence** and feeling unable to reduce or cessation, relying on BZRA for comfort and feeling unable to sleep without it **17 [15,16,17,19]**	**Expected patient resistance** towards deprescribing of BZRA **7** [20]	**Unequal balance of power** between nurses and physicians, including nurses feeling their options are not considered or valued **8** [22]
**Theme** **Frequency [ref.no.]**	Discouragement or **lack of support** from physician **4** [17,18]	**Reluctance to deprescribe** treatment from functioning patients **2** [20,21]	General attitude that BZRA should not be avoided and **continued use is necessary** **1** [22]
**Individual Facilitators**			
	**Patients**	**Physicians**	**Nurses**
**Theme** **Frequency [ref.no.]**	The patients are **willing to stop** BZRA treatment **2** [8,19]	The physicians know and **agree with guidelines** instructing that BZRA is only for short-term use **1** [20]	**Involving the nurses** in the patient´ medications and evaluation of medications can encourage the nurses to facilitate deprescribing **2** [22]
**Theme** **Frequency [ref.no.]**	Mentioning deprescribing to patients and giving the patient **time to consider** the benefits **2** [8,17]	No data	Systematic **work procedures** to record their observations on patients’ sleep **2** [22]

## References

[1] Matheson E., Hainer B.L. (2017). Insomnia: Pharmacologic Therapy. Am. Fam. Physician.

[2] Pottie K., Thompson W., Davies S., Grenier J., Sadowski C.A., Welch V., Holbrook A., Boyd C., Swenson R., Ma A. (2018). Deprescribing benzodiazepine receptor agonists: Evidence-based clinical practice guideline. Can. Fam. Physician..

[3] Vinkers C.H., Olivier B. (2012). Mechanisms Underlying Tolerance after Long-Term Benzodiazepine Use: A Future for Subtype-Selective GABA(A) Receptor Modulators?. Adv. Pharmacol. Sci..

[4] Ng B.J., Le Couteur D.G., Hilmer S.N. (2018). Deprescribing Benzodiazepines in Older Patients: Impact of Interventions Targeting Physicians, Pharmacists, and Patients. Drugs Aging.

[5] Donoghue J., Lader M. (2010). Usage of benzodiazepines: A review. Int. J. Psychiatry Clin. Pr..

[6] Smith A.J., Tett S.E. (2009). How do different age groups use benzodiazepines and antidepressants? Analysis of an Australian administrative database, 2003–2006. Drugs Aging..

[7] Fick D.M., Semla T.P., Steinman M., Beizer J., Brandt N., Sandhu S., 2019 American Geriatrics Society Beers Criteria^®^ Update Expert Panel (2019). American Geriatrics Society 2019 Updated AGS Beers Criteria^®^ for Potentially Inappropriate Medication Use in Older Adults. J. Am. Geriatr. Soc..

[8] Chen L., Farrell B., Ward N., Russell G., Eisener-Parsche P., Dore N. (2010). Discontinuing Benzodiazepine Therapy: An Interdisciplinary Approach at a Geriatric Day Hospital. Can. Pharm. J..

[9] Stern C., Jordan Z., McArthur A. (2014). Developing the Review Question and Inclusion Criteria. Ajn Am. J. Nurs..

[10] Det Kongelige Bibliotek (2020). Københavns Universitetsbibliotek. Sundhedsvidenskab: Farma. https://kub.kb.dk/sund/farma.

[11] (2017). EndnoteTM.

[12] Robson C., McCartan K. (2016). Real World Research.

[13] (2020). Nvivo.

[14] Tong A., Sainsbury P., Craig J. (2007). Consolidated criteria for reporting qualitative research (COREQ): A 32-item checklist for interviews and focus groups. Int. J. Qual. Health Care.

[15] Canham S.L., Gallo J., Simoni-Wastila L. (2014). Perceptions of Benzodiazepine Dependence Among Women Age 65 and Older. J. Gerontol. Soc. Work..

[16] Heser K., Pohontsch N.J., Scherer M., Löffler A., Luck T., Riedel-Heller S.G., Maier W., Parker D., Haenisch B., Jessen F. (2018). Perspective of elderly patients on chronic use of potentially inappropriate medication–Results of the qualitative CIM-TRIAD study. PLoS ONE.

[17] Martin P., Tannenbaum C. (2017). A realist evaluation of patients’ decisions to deprescribe in the EMPOWER trial. Bmj Open.

[18] Tannenbaum C., Martin P., Tamblyn R., Benedetti A., Ahmed S. (2014). Reduction of inappropriate benzodiazepine prescriptions among older adults through direct patient education: The EMPOWER cluster randomized trial. JAMA Intern. Med..

[19] Williams F., Mahfouz C., Bonney A., Pearson R., Seidel B., Dijkmans-Hadley B., Ivers R. (2016). A circle of silence: The attitudes of patients older than 65 years of age to ceasing long-term sleeping tablets. Aust. Fam. Physician.

[20] Cook J.M., Marshall R., Masci C., Coyne J.C. (2007). Physicians’ Perspectives on Prescribing Benzodiazepines for Older Adults: A Qualitative Study. J. Gen. Intern. Med..

[21] Šubelj M., Vidmar G., Švab V. (2010). Prescription of benzodiazepines in Slovenian family medicine: A qualitative study. Wien. Klin. Wochenschr..

[22] Anthierens S., Grypdonck M., De Pauw L., Christiaens T. (2009). Perceptions of nurses in nursing homes on the usage of benzodiazepines. J. Clin. Nurs..

[23] Pickering A.N., Hamm M.E., Bs A.D., Hanlon J.T., Thorpe C.T., Gellad W.F., Radomski T.R. (2020). Older Patient and Caregiver Perspectives on Medication Value and Deprescribing: A Qualitative Study. J. Am. Geriatr. Soc..

[24] Reeve E., Shakib S., Hendrix I., Roberts M.S., Wiese M.D. (2014). Review of deprescribing processes and development of an evidence-based, patient-centred deprescribing process. Br. J. Clin. Pharm..

[25] Scott I.A., Hilmer S.N., Reeve E., Potter K., Le Couteur D., Rigby D., Martin J.H. (2015). Reducing inappropriate polypharmacy: The process of deprescribing. JAMA Intern. Med..

[26] Kua C.-H., Mak V.S., Lee S.W.H. (2019). Perspectives of health professionals towards deprescribing practice in Asian nursing homes: A qualitative interview study. BMJ Open.

[27] Hoffmann F. (2013). Perceptions of German GPs on benefits and risks of benzodiazepines and Z-drugs. Swiss Med. Wkly..

[28] Heinemann S., Brockmöller J., Hagmayer Y., Himmel W. (2019). Why Z-drugs are used even if doctors and nurses feel unable to judge their benefits and risks—A hospital survey. Eur. J. Clin. Pharm..

